# Ideal Workers, Supporting Actors, or Thrill Seekers? How Coworker Demands Influence Ambulance Volunteers’ Experiences of Freedom and Meaningful Work

**DOI:** 10.1007/s11266-024-00690-3

**Published:** 2024-10-22

**Authors:** Kirstie McAllum

**Affiliations:** Department of Media and Communication, University of Canterbury, Private Bag 4800, Christchurch, New Zealand

**Keywords:** Volunteers, Freedom, Meaningful work, Meaningfulness, Volunteer management

## Abstract

For nonprofit organizations (NPOs) struggling to attract adequate numbers of volunteers, examining what makes nonprofit engagement meaningful is essential because disenchanted volunteers can simply quit. Yet, the assumption that freedom is a core aspect of the volunteer experience and of meaningful work may not hold true in high-stakes environments where volunteers must demonstrate high levels of commitment and expertise. This study aims to analyze how freedom plays out in high-stakes volunteering and its impact on meaningful work. Drawing on interviews with volunteer and paid ambulance crew working in nine stations in Aotearoa New Zealand, the study explores how “super-volunteers” talk about freedom in the context of their on-road work and how coworkers communicatively attempt to influence volunteers’ freedom. Three volunteer profiles emerged from the analysis: ideal workers, supporting actors, and thrill seekers. Most paid staff encouraged ideal workers to strive for self-realization, a form of positive freedom *in* work, which led to optimal clinical performance. Supporting actors privileged self-determination or positive freedom *at* work, although coworkers successfully pushed them to contribute to basic emergency work. Because thrill seekers demanded freedom *from* boring or dirty jobs, appeals to teamwork failed to sway them. The study makes two key contributions. First, the diversity of freedoms volunteers evoked and resisted underscores the importance of nuancing the assertion that volunteering is a “free” act. Second, although the meaningful work literature is drifting in the pro-freedom direction, it shows that the freedoms enacted by volunteers or promoted by coworkers were arguably “mistaken”—for volunteers, patients, and the NPO itself.

**Supplementary Information:**

The online version contains supplementary material available at 10.1007/s11266-024-00690-3.

Despite the efforts of taskforces created to boost volunteer numbers, scholars and practitioners who study nonprofit organizations (NPOs) express dismay at the shrinking volunteer workforce (see Forner et al., 2024). Some analysts attribute the shortage to increased workforce participation, work intensification, and employer-mandated corporate volunteering, which create staunch “competition from other greedy institutions” (Brudney & Meijs, 2009, p. 570). Others point to an upsurge in the number of “reflexive” volunteers who select short-term projects that interest them but drop out when their enthusiasm wanes (Hustinx & Lammertyn, 2003; Hyde et al., 2016). In a context where volunteer labor is scarce, examining what makes nonprofit engagement meaningful is vital because disenchanted volunteers who find causes or assignments meaningless can simply quit (Brudney, 2016).

How freedom plays out in decisions to dis/engage is of interest to scholars of both volunteering and meaningful work. The volunteering and volunteer management literature presents autonomy, free will, choice, or lack of coercion as an essential element of the volunteer experience (e.g., Cnaan et al., 1996; McAllum, 2014; Smith et al., 2017). Likewise, research on meaningful work highlights the “centrality of freedom to the experience and achievement of meaningful work” (Breen, 2019, pp. 51–52) and numerous empirical studies have “modelled and empirically confirmed that autonomy is an antecedent of MW [meaningful work]” (Both-Nwabuwe et al., 2020, p. 105). Because individual tastes vary, it is unsurprising that most popular and scholarly accounts of meaningfulness insist that what counts as fulfilling work is highly subjective (Rosso et al., 2010). What is aptly named the “self-realization” perspective of meaningful work (Lepisto & Pratt, 2017) assumes that if individuals are “the ultimate arbiter of the meaning of their own work, meaningfulness becomes self-referential” or “meaningful for me” (Michaelson, 2021, p. 416). From this perspective, NPOs wishing to attract and retain volunteers must cater to their preferences.

Yet, volunteers’ ability to make “frequent entries and withdrawal” (Hustinx & Lammertyn, 2003, p. 175) as they see fit may collide with NPO demands and/or beneficiaries’ needs, especially in high-stakes environments like emergency services. As McNamee and Peterson (2016) contend, NPOs whose mission involves “high risk activities requiring extensive training (…) may find it logistically and financially impossible to sustain their efforts” (p. 276) with “revolving door” or “plug-in USB key” volunteers (Eliasoph, 2009). Given the importance of reliability, high-stakes NPOs want to attract “super-volunteers” who contribute more than ten hours per week to an NPO (Einolf & Yung, 2018) because these volunteers will turn up regularly, develop expertise, and contribute to organizational mission long-term.

Previous studies have shown that NPOs can design volunteer roles and supervisory practices so that volunteers deploy their freedom in line with organizational expectations (e.g., Hustinx et al., 2008). However, the ways in which other organizational members, such as coworkers, try to cultivate and curtail volunteers’ freedom have been overlooked. By analyzing organizational others’ influence on how high-stakes ambulance volunteers experienced freedom, this study makes several contributions. First, rather than viewing meaningful work exclusively through a self-realization lens that privileges subjective desires and decisions, it insists that freedom is multiform and relational. Second, it problematizes the assumption that freedom contributes positively to meaningful work. The forms of freedom enacted by volunteers and promoted by organizational others also generated outcomes that threatened the meaningfulness of volunteers’ contribution.

The literature review which follows documents how NPOs account for and attempt to “manage” volunteers’ freedom, before theorizing the notion of freedom itself and assessing its consequences for meaningful work. Next, I situate the research context and describe data collection and analysis strategies, before turning to the findings and implications for theory and practice.

## Literature Review

Classic definitions of volunteering list freedom as a sine qua non for an activity to be voluntary or carried out with free will. Meaningful work scholars, for their part, contend that individuals must experience some latitude in what their work is and how they do it if they are to flourish. Given the centrality of freedom to both streams of research, it is essential to delve into how freedom is conceptualized and how it contributes to meaningful work.

### Freedom, Volunteering, and Volunteer Management

Cnaan et al.’s (1996) oft-cited definition describes volunteering as a “free” act that contributes to the public good, is conducted primarily in structured organizational settings, and requires the personal costs of volunteering to exceed the benefits received. Musick and Wilson (2008) understand freedom as an individual decision to initiate or continue performing a desirable action. Experts in volunteer management draw on these meanings of freedom when they advise NPOs to make volunteering (more) attractive by highlighting what volunteers’ efforts enable the organization or beneficiaries to achieve; instigating recognition activities and awards; offering training and professional development opportunities; and creating more interesting organizational assignments (e.g., Brudney, 2016). Other definitions present freedom as the ability to choose among competing options without constraint. Practitioners adopt this perspective when they provide a loosely structured organizational environment and accept flexibility rather than insisting on commitment.

At times, advice on managing volunteers interprets freedom more ambiguously. “Winning volunteer scenarios,” volunteer management researchers Meijs and Brudney (2007) argue, require an AAA approach, whereby managers align volunteers’ *assets*, their *availability*, and organizational *assignments*. This strategy may seem, at face value, to integrate both dimensions of freedom discussed above. NPOs may emphasize a role’s desirability (assignment) and accept the talents (assets) and time (availability) that volunteers offer without forcing them into a one-size-fits-all mold. By contrast, NPOs may subtly limit volunteers’ freedom by allowing them to select tasks (assignment) that align with their interests (assets) and calendar (availability) from among a predetermined, highly structured set of activities (Hustinx et al., 2008). In this way, as Hustinx (2010) notes, “individual modes of action are simultaneously *favoured* and *enforced*” (p. 168, italics in the original). Volunteers may believe they can choose without constraint, but NPOs control when and how freedom is exercised.

Across this set of definitions, freedom takes on quite distinctive meanings. Surprisingly, NPO scholarship lacks a robust conceptual apparatus to parse out the various forms of freedom that remain implicit in volunteer management praxis and theorization. The meaningful work literature, by contrast, has explored this question in depth.

### Freedom and Meaningful Work

The concept of freedom is multi-faceted: according to Isaiah Berlin (1969), a political philosopher, “the meaning of this term is so porous that there is little interpretation that it seems able to resist” (p. 121). To delineate freedom’s conceptual reach, Berlin differentiates negative from positive freedom, a distinction that continues in more recent typologies (e.g., Carter, 2022; Veltman, 2016). According to Berlin (1969), negative freedom refers to the scope of action within which an individual “is or should be left to do or be what he is able to do or be without interference by other persons” (pp. 121–122) (e.g., a volunteer questions a patient without their more qualified coworker taking over). Negative freedom may, however, be curtailed in the name of other values, such as security, solidarity, or equality. Positive freedom, or the freedom to decide which activities to undertake or what kind of life to lead, is present when individuals exercise self-mastery or self-governance (Berlin, 1969) (e.g., a volunteer chooses to help a paid coworker with the drug count). In sum, if negative freedom focuses on “how much authority” individuals wield and seeks to eliminate external obstacles that curb independence, positive freedom considers “whose authority” guides decision-making. Surprisingly, Berlin (1969) insists that positive freedom is compatible with manipulation when self-mastery is equated with the development of one’s rational, autonomous, “best” self. That is, governments or employers may “promote” positive freedom in authoritarian ways by disciplining unenlightened lower passions and practices that impede attainment of more transcendent goals (Breen, 2019; Ciulla, 2012).

Despite positive freedom’s potentially coercive tendencies, meaningful work scholarship embraces it as a dominant organizing framework, viewing positive freedom in terms of (1) self-realization and (2) self-determination. Self-realization clearly maps onto accounts of meaningful work that privilege individual flourishing, subjective fulfillment, and engagement in personally significant work (Lysova et al., 2019; Michaelson, 2021) (e.g., the volunteer assisting with the drug count finds it interesting and/or important, and therefore meaningful). The bulk of the meaningful work literature advances the argument that all subjective interpretations of self-realization constitute “genuine claims to meaningful work” and are therefore “equally valid” (Michaelson, 2021, p. 417). Roessler (2012) explains that if, as liberal theorists argue, the value pluralism present in liberal democracies means that citizens do not share common values, workers must pursue meaningful work as they see fit. Self-determination, the second aspect of positive freedom, arises when workers with an internal locus of control pursue intrinsically motivating jobs that align with their life goals and design and organize their work environment to achieve them (Both-Nwabuwe et al., 2020). Here, positive freedom generates authenticity, self-concordance, and self-connection, all of which contribute to meaningful work (Lips-Wiersma & Morris, 2017; Rosso et al., 2010).

These accounts of positive freedom pay scant attention to constraints on negative freedom. Even theorists who explicitly tackle negative freedom tend to describe it in ways that resemble positive freedom. For example, scholars who criticize Berlin’s (1969) view of negative freedom as noninterference contend that non-domination is what would allow all workers—and not just those with lenient, laissez-faire, or enlightened bosses—to experience freedom (Breen, 2019; Pettit, 1997).^1^ Noninterference, they argue, only offers workers a temporary reprieve from others’ arbitrary meddling in how they organize and conduct their work (e.g., coworkers do not interrupt volunteers’ patient questioning during straightforward jobs), whereas non-domination is enduring (e.g., coworkers never interrupt when volunteers are “leading” a job). Yet, non-domination affords workers self-mastery or self-determination: a form of positive freedom (e.g., volunteers choose to initiate patient questioning).

A much smaller stream of research assesses how positive and negative freedom intersect. Conceptually, meaningful work scholars argue that negative freedom, understood as the absence of structural, contextual, and interpersonal barriers or obstacles, is a prerequisite for positive freedom (Carter, 2022; Ciulla, 2012). Ciulla (2012) designates negative freedom as “the condition needed to find meaningful work” and positive freedom as “the content of meaningful work, or what makes work meaningful for people” (p. 118). Michaelson’s (2021) analysis of young Chinese working on a tour boat on the Yangtze provides a fascinating example of a conflict between the two. When one over-confident porter promotes himself as a second-rate singer to earn tips from wealthy tourists (exercising his positive freedom), his supervisor crushes the initiative by firing him (limiting his negative freedom). This body of work presumes that although workers decide what positive freedom means for them, more powerful organizational others can constrain negative freedom.

These assumptions may not hold true for super-volunteers: NPOs may fear upsetting volunteers who constitute the mainstay of their workforce. Such was the case for professionally savvy super-volunteers in Einolf and Yung’s (2018) study, who demanded discretionary decision-making power and resisted being “robotic” or made “to follow (…) on an apron string” (p. 802). In contexts with tightly regulated occupational practices, however, managers and coworkers may not treat super-volunteers with kid gloves but accuse those who wish to be “autonomous agents capable of undertaking their own course of action” (Hustinx, 2010, p. 168) of excessive individualism. I argue that meaningful work scholarship needs to pay more attention to how these organizational others—in this case, super-volunteers’ paid and unpaid coworkers—provide important cues for how volunteers interpret both forms of freedom. Accordingly, I ask the following research questions:

#### RQ1

How do super-volunteers in a high-stakes NPO talk about freedom in the context of their core role?

#### RQ2

How do coworkers attempt to influence how super-volunteers enact freedom?

## Method

The data for this paper comes from a larger cross-sectional study on ambulance crews’ role negotiations, conducted in partnership with Hato Hone/St John Ambulance (henceforth, St John), Aotearoa New Zealand’s largest NPO. St John provides emergency ambulance services to 97% of the population of five million. Government funding covers paid staff salaries, but, in areas with low or dispersed populations, volunteers work alongside other volunteers or paid staff to ensure double crewing^2^ (there were 2,912 on-road volunteers to 1,567 paid staff in 2022). St John insists that, since all organizational members are “health professionals,” paid staff and volunteers wear the same uniform and receive the same training. Training begins at First Responder level (basic first aid) and continues through the base ambulance officer qualification of Emergency Medical Technician (EMT) to Paramedic and Intensive Care Paramedic (ICP).

### Data Collection and Analysis

The broader project mobilized three types of qualitative data: 85 h of recordings of observations at 12 ambulance stations (three all-paid, three all-volunteer, and six “mixed” volunteer/paid stations); 147 single-spaced pages of field notes about on-road interactions; and 898 pages of interview data. Most of the project’s 120 participants elaborated on their role during “ethnographic” interviews (informal conversations that occurred during observations). Fifty-four (27 volunteers and 27 paid crew) participants elected to participate in more formal, semi-structured interviews about paid staff/volunteer roles, responsibilities, and relationships (questions included “What are your responsibilities on-road?”; “What is the role of paid staff/volunteers? What are they *not* responsible for?” and “What jobs should they do that they don’t do? How do you manage this?”; see Supplementary File 1 for the complete interview guide). Interviews varied in length: Some lasted two to three hours while emergency calls cut others short after eight minutes (*M* = 42 min). This study focuses on semi-structured interview and observational data from the nine stations where volunteers were present.

To answer the first research question, *How do super-volunteers in a high-stakes NPO talk about freedom in the context of their core role?*, I read volunteers’ transcripts multiple times and highlighted passages where they described what their role entailed. I then engaged in an iterative analysis that “alternate[d] between emic, or emergent, readings of the data and an etic use of existing models, explanations, and theories” (Tracy, 2020, p. 209), linking first-level emic codes with different forms of positive and negative freedom (e.g., “catching the bug” with self-realization; “assisting paid staff” and “attending ‘good’ jobs” with self-determination; and “sidestepping boring jobs” with negative freedom).

I then grouped codes into broader categories that reflected how different groups of volunteers talked about freedom (see Supplementary File 2 for an example of the coding process). Because St John benefitted from the growing skillfulness of volunteers who enthusiastically threw themselves into complex jobs, I named volunteers for whom self-realization was key “Ideal Workers.” By contrast, volunteers whom I called “Supporting Actors” used self-determination to limit their role to simpler or nonclinical tasks. Self-determination manifested quite differently for “Thrill Seekers,” skilled volunteers who only attended interesting jobs and who fobbed off coworker demands using negative freedom.

To address the second research question, *How do coworkers attempt to influence how super-volunteers enact freedom?*, I analyzed coworkers’ reactions to volunteers’ enactment of self-realization, self-determination, and negative freedom. Coworkers responded very differently to each. By normalizing ideal workers’ dedication (“cultivating clinical competence”; “insisting on the need to earn their ATP level”), most coworkers reinforced volunteers’ desire for self-realization, although a few encouraged volunteers to cut back. Some coworkers accepted supporting actors’ narrow role interpretation, but most contested volunteers’ self-determination by “stressing emergency preparedness.” Coworkers unsuccessfully contested thrill seekers’ negative freedom by appealing to values like “solidarity” and “teamwork.”

### Findings

The findings describe how each volunteer profile—ideal workers, supporting actors, and thrill seekers—talked about freedom (RQ1) before turning to coworkers’ attempts to influence how each group enacted freedom (RQ2).

#### Ideal Workers’ Drive for Self-Realization

Volunteers who fit the ideal worker profile referred to ambulance work as exciting, stimulating, and significant, descriptions that resonate with a *self-realization* take on positive freedom. Many talked about “having the bug,” “being hooked,” “being keen as mustard,” and “loving” their volunteer role. Volunteers mentioned, “I loved it right from when I started, you know? It’s been really cool;” “Bug’s like, everyone gets it, because otherwise you wouldn’t do it;” and “Volunteers have got a bug-we call it the bug, so you get really passionate and get energized by it and also this is a new thing, where there’s lights, there’s sirens.” According to a volunteer station manager, 80% of her volunteers have “the bug.”

Ideal workers’ “passion for working on the road” translated into a desire to sharpen their knowledge and attain a higher authority to practice (ATP)^3^ by getting fully involved in complex on-road jobs. These volunteers spoke about “chasing calls” and wanting to get as many jobs as they “could get their hands on” “under their belt” for the national EMT diploma or paramedic qualification. Their “eagerness” led volunteers to increase their on-road time far beyond the mandated minimum of one shift per fortnight. Examples abounded:

I’m only here [at the station] two days a week for sort of 12–18 hours. I try and do a weekend night or a weekend day and a night during the week. (Volunteer first responder)

I’m usually at four [12-hour shifts] a month. Yeah, so I do every Wednesday, sorry six a month because I tend to do every second weekend as well. (Volunteer EMT)

One rural first responder explained that “I sign on when I’m available, and (…) so really except for the 40-h [paid] work week, if I’m not out socializing, I’m basically signed on [paged when a medical emergency or car crash occurred in the area].” An ideal worker with no intention of seeking a paid position remarked that “career volunteers” who aspired to “use” volunteering as a springboard for employment “will do a lot more to show that they can do all these things, show that they’re keen.”

When self-realization led volunteers to view their St John role as a central, highly meaningful life interest, they frequently prioritized volunteering over family and other leisure commitments. For instance, during a conversation with a fellow volunteer, a first responder recounted that his children were complaining that he always slept during the day to recuperate from nightshifts. Yet, when the other volunteer “made it clear that he needed to cut back on shifts,” the field notes infer that “there was almost a reluctance from the first responder to agree.”

#### Coworkers: Pressuring Ideal Workers to Perform Perfectly

Nearly all-paid coworkers normalized volunteers’ “ideal worker” practices because only through continuous clinical upskilling could volunteers be truly useful. Paid staff cultivated clinical competence by reinforcing the need to study and complimenting volunteers who did so. During an observation, a paid EMT commented to a first responder reading a manual, “Look at you, you are so studious. Volunteers setting a good example!” Observations showed paid coworkers taking the initiative, as when an ICP quizzed a volunteer EMT about a particular case. The field notes read: “The volunteer is receptive to this, generates some answers, and begins to learn from the ICP.” Some stations’ physical environment confirmed study’s importance: “the bookshelf is full of educational books on anatomy and first aid. A practice dummy is noticeably visible on the desk as you enter the room.” Overall, this monitoring of volunteers’ study highlighted the “expectation that they’re being watched as well, that it’s not a free ride.”

Paid coworkers also pushed volunteers, particularly those with an EMT patch, to perform their role as paid staff would by mentioning identical training opportunities. A paid EMT argued that “we’re all taught the same thing at CCE [Continuing Clinical Education] training. We’ve all got the same qualification. We’ve all been through the same training, so if they’re [volunteers] a bit rusty on anything, they shouldn’t be.” Consequently, paid coworkers criticized under-performance:

It makes no difference whether you’re paid or volunteer. I think that if you’re a health professional-and the word professional is probably the most important bit-you come to work with a professional attitude, and you perform to the level of skill that’s expected of you. So, I believe that an EMT volunteer should be performing to the same level medically as an EMT paid. There may be some things they do less of so they might not be sure of things, but if they have a qualification on their shoulder, that’s their job at that time.

If volunteers were not “willing to act in accordance with their ATP,” some paid staff concluded that “they should be on as a third [person, an observer]. They’re not just there to keep the seat warm.”

For most ideal workers interviewed, coworker pressure consolidated their desire for self-realization through skill development. According to a volunteer EMT, “We have to have the same standard as a paid member. We don’t get anything less just because we’re not getting paid. We have to do all the same tests, qualify, and maintain all the same protocols as they do.” Another volunteer EMT corroborated that “once you get the EMT [patch], they [paid staff] expect you to be able to do certain things, so to be able to keep up and do those things, it challenges you and keeps you sharp.” For many volunteers, performance expectations meant that when they were not out on a job, “I try and spend as much time as I can studying, or just talking about things with the paid officers that are there.”

Yet, coworker pressure could also make self-realization less attractive, and as it ebbed, self-determination, or the ability to enact the role in less demanding ways or step away from it entirely, became more important. A rural volunteer EMT explained that when she landed at an urban station due to fluid deployment,^4^ she preferred to relax rather than refresh her clinical knowledge:

When I’m on station, it’s a very good time for me to do my emails. Sometimes you can ask to do a bit of clinical work with one of the paid staff, just go through a cardiac arrest or whatever, something you want to brush up on. I personally hate doing stuff like that and even though these people [paid staff] on Station Y that think while we’re [volunteers] at the station we should be doing things, I quite like to read my book actually.

Other volunteers with decades of experience mentioned that although they were “initially” “as keen as mustard and right into it,” they had thought about “stepping back” or dropping out because the training and time requirements had become overly onerous. These old hands mentioned that EMT skill requirements had ballooned over the years (“now they [St John] are trying to get people to be paramedics, that’s where they want everyone to be”’; “St John, it’s just so demanding (…) you have to give it 120%”), which put “more pressure on” and made them feel “less secure” in their practice. Another volunteer EMT reflected, “what I’ve found over the last 20 years is that there are so many more things they [St John] expect you to know, to be able to do, but they must be getting to the stage where this is untenable.”

#### Coworkers: Encouraging Ideal Workers to Slacken Off

Given the large number of participants whose St John involvement aligned with the ideal worker profile, it is surprising how few coworkers commented on its potential dark side, as though this way of working were normal. Some paid coworkers, notably station managers and volunteer coordinators, tried to convince “overly excited volunteers” to reduce their shifts. A paid manager recalled that when he started as a volunteer, “you just want to give it your all, don’t you? Well, you do. They [volunteers] just want to give all their time (…) I did it myself, said no to nothing, said yes to everything.” The bug was dangerous because it led volunteers to become “a yes man, like ‘Yes, I’ll do this shift,’ ‘Yes, I’ll do that,’ (…) ‘I’m going to come in five days a week,’ ‘I’m going to do 12-h shifts and I’m going to max my hours out.’” Some paid staff mentioned that the effort and time investment could lead to burnout after a few years: “You end up working yourself to the bone and then you end up losing your motivation, like it just drops off and as a result you lose a volunteer.” Interestingly, these paid staff focused on the number of shifts but did not mention the risk of previously enthusiastic volunteers becoming jaded by demands for continuous study and training.

#### Supporting Actors’ Urge to Take a Back Seat

Supporting actors were volunteers who whittled their role down to something they were comfortable with. They interpreted freedom in terms of *self-determination*, a positive freedom whereby people select roles adapted to personal goals. They viewed their role as “helping” coworkers by being an “assistant” who could “fetch and carry” bags and equipment, and “lift, drive, and carry stuff.” Other supporting actors limited their role to “helping with the basics.” To align the on-road environment with this vision, they expected to be excused from complex clinical work, a decision that forced more qualified coworkers to take charge of medical treatment. For example, a first responder who reported being afraid of needles following heart surgery hung back while the paid paramedic on the truck administered morphine.

#### Coworkers: Spurring Supporting Actors to Pick up the Pace

Some coworkers accepted volunteers’ (and particularly first responders’) wish to be supporting actors. A paid rural paramedic affirmed, “That’s fine, we have no problem with that because it’s another set of hands onboard (…) and it’s another shift that’s covered, so the roles vary.” Another paid coworker described supporting actors as

quite happy just to drive out. They’re not into it for the glory or improving themselves or anything like that, they’re just happy as a volunteer in this role, doing what they do, which is brilliant. We need those people, they’re rocks.

Similarly, an urban EMT explained that even first responders uninterested in taking their learning further “stop me from being in danger single crewed, so I just let them do what they want to do, and when we get a job, as long as they come with me, I’m happy.”

However, most coworkers constrained supporting actors’ ability to view their role in reductive ways by stressing the importance of developing “basic” on-road knowledge. An urban station manager enumerated a long list of skills that “brand spanking new and green” first responders should have: take blood pressure and pulse, set up equipment for measuring heart rate, and locate supplies. Another paid paramedic criticized first responders who “don’t really take responsibility for their own learning:”

They might have been at a station for a period of time, and they still don’t know how to do very, very basic tasks. They say they haven’t been shown, but they’ve seen a paramedic or an EMT do it a million times, and I’m pretty sure they *have* been shown, because they have a qualification on their shoulder that says they’re a first responder. But yeah, not having the confidence to do it or wanting to step up to do it.

Paid coworkers justified these demands, insisting that, because patients’ lives hung in the balance, volunteers couldn’t say “‘But I’m only a volunteer!’ That doesn’t wash with the patient.” From their perspective, “even though it’s a volunteer role, it’s not a freedom. It’s not a way to get out of stuff. That’s not acceptable.”

In a similar vein, when supporting actors seemed reluctant to conduct pre-shift vehicle checks, paid crew frequently gave them a nudge by invoking emergency preparedness:

If they go “Oh, I don’t know…”, I’ll say “It’s good for you to have the exposure in our new truck where everything is. If we do get a Status 1 [life-threatening situation], then at least you don’t go flustering.” And a lot of time, when I tell them “Status 1,” they go, “Yes, okay, I’ll come.”

In one particularly telling example, a paid paramedic got “sick of a volunteer who would turn up just in time for shift to start and come down and have a coffee.” When the volunteer continually gave no more than a cursory glance over the ambulance despite her urging him to become familiar with the equipment by doing the check, she changed tactic:

He would disappear for two minutes (…) so Week 4, I took something out. Well, I didn’t take it out: I moved it, so he might not see it. And I said, “You need to go and do the vehicle check.” So, he comes back, and I go, “All good?” And he goes, “Yeah.” I said, “Anything missing?” “No.” “Really?” And he goes, “I’ll be back in a minute.” Ever since then, he was there ten minutes early, he was in the truck with me, and he was checking the truck.

Importantly, several participants noted that such tactics only worked when paid staff conducted the check with volunteers. Paid staff who sent volunteers off alone engendered resentment and resistance.

#### Thrill Seekers’ Penchant for Exciting Jobs

Self-determination manifested itself quite differently for a small number of first responders and volunteer EMTs who were “thrill seekers.” Because this group viewed their role as engaging in interesting emergency response work, they unilaterally decided which jobs they would attend. The dataset contains several instances of volunteers refusing to go to “boring” or “dirty” jobs involving diarrhea or vomiting, insisting it was not their job to “clean up sick.”

Interestingly, volunteer and paid coworkers criticized thrill seekers. A rural volunteer complained when crew from her station, already on route to a poorly staffed neighboring village, received messages that the village crew would, in fact, attend the job:

I mean, they’re either on duty or they’re not (…) And the worst thing is, I get the impression a lot of them look at it and think “Oh yes, that will be an interesting job, so I’ll ring up and say we’re available.” (…) So as a volunteer, I’m annoyed at those other volunteers [laughs].

The chief complaint was that thrill seekers who were “half in and half out [of St John]” cherry-picked “good” jobs.

Both paid and volunteer coworkers also expressed feeling “frustrated” by thrill seekers’ insistence that nobody could question their choices. Negative freedom, or freedom from interference by others, expressed itself in the “attitude” of “‘Well I don’t get paid for what I do, so I’ll do what I like. You can’t fire me.’ (…) It’s just the undertones, like say if there’s something going on, they’ll say, ‘Oh well, you know, I *am* just volunteering.’” A paid EMT attributed refusals to volunteers’ freedom, noting “they are able to kind of not do it, and it’s a freedom that they are afforded.” A paid paramedic likewise noted, “I think some of them use it [their status] as an excuse, a little bit. They use it as an excuse. ‘I’m just a volunteer so I don’t *need* to do this.’”

#### Coworkers: Inciting Thrill Seekers to Act as Team Players

Coworkers had few strategies to deal with thrill seekers’ reasoning that “because you’re a volunteer, you have an excuse to get out of things.” Whereas paid coworkers could push back against supporting actors’ choices by appealing to patient safety as a “higher” value, they could not do so with thrill seekers who did, in fact, attend “real” emergencies. Coworkers unsuccessfully tried to deal with apathy or resistance by volunteers who “don’t do things off the cuff” and who were “there for a ride, (…) to muck around” by highlighting other values such as solidarity and teamwork. A paid EMT insisted that “You can’t be part of the team for just the good things. You’ve got to be part of the team for everything.” Others articulated “the view that, ‘You’re still part of the team, so you should contribute just as much,’” and “Oh, you’re here volunteering. You’re one of us. We should all be doing the same amount.”

## Discussion and Conclusion

This study set out to explore how super-volunteers talked about freedom in the context of their core role and how coworkers tried to influence their enactment of freedom. The findings show that volunteers understood freedom in terms of self-realization and self-determination, both forms of positive freedom, as well as negative freedom. Yet, freedom was not solely an individual endeavor but relational; coworkers influenced, to some extent, how freedom was enacted. Nearly all-paid staff encouraged ideal workers to strive for self-realization *in* their work, because it contributed to optimal clinical performance. For supporting actors, self-determination took the form of “autonomous agency” (Veltman, 2016, p. 82) or positive freedom *at* work, although coworkers successfully pushed them to contribute to basic emergency work. Because thrill seekers, who expected coworkers to respect their scope of action (negative freedom), demanded freedom *from* boring or dirty jobs, appeals to teamwork failed to sway them.

These findings make two key contributions. First, the diversity of freedoms volunteers evoked, enacted, and resisted underscores the importance of nuancing the assertion that volunteering is a “free” act (Cnaan et al., 1996; McAllum, 2014; Smith et al., 2017). A range of positive and negative freedoms co-existed with organizational and coworker expectations that limited volunteers’ freedom. As Lindebaum et al. (2022) point out, “freedom is not experienced in terms of either/or, as either present or absent, but as a hybrid of (un)freedom: the experience of simultaneously being free and unfree” (p. 1862). Moreover, (un)freedom can take a multiplicity of forms. Fleming (2022), for instance, proposes that freedom at work can be split into contractual, spatial–temporal, professional, and vocational freedoms. Although volunteers sign on for at least one shift per fortnight, they may pull back or drop out anytime, giving them extensive contractual freedom. Tight rules regulating authority to practice limits volunteers’ professional freedom on road, offering more qualified coworkers greater scope to push them to perform, yet volunteers enjoy significant spatial–temporal freedom. They can choose which shifts they will do, decide whether they will go to jobs or not, and during jobs, how they will position themselves with respect to the patient, the patient’s family, and other crew (McAllum, 2020).

When volunteers do not find prescribed work meaningful, it is not necessarily the case that other organizational members or the organization itself does not respect their freedom, but that they are fostering the wrong “type” of freedom. By showing how freedoms are present or absent in multiple forms of varying intensity, this study contributes to meaningful work research that seeks to go beyond the idea that meaningfulness is a static, globally positive attitude toward one’s job that can grow (Lysova et al., 2022). Second, although the meaningful work literature is drifting in the pro-freedom direction, this study shows that freedom enacted by volunteers and promoted by coworkers could be “mistaken” on several counts (Michaelson, 2021). Coworkers’ attempts to influence volunteers’ freedom point to a belief that subjectively meaningful work can be “wrong” and “potentially harmful-when it motivates [workers] to engage in (…) work that is unproductive, counterproductive, or even destructive” (Michaelson, 2021, p. 418). Rather than privileging subjective meaningfulness, paid coworkers’ (re)actions align far better with social accounts of meaningfulness or what Lepisto and Pratt (2017) call the “justification perspective” of meaningful work. Instead of relying on individual sense-making, the social recognition perspective links meaningfulness to *others’* evaluations of a job’s worth and its quality (Michaelson, 2021).

Many coworkers insinuated that supporting actors and thrill seekers, who valued role elasticity and freedom from formal constraints, were on a pathway to failure as ambulance volunteers. While the volunteer management literature advises NPOs to respect and even encourage freedom by offering flexible, volunteer-centric assignments (e.g., Brudney, 2016), most paid coworkers deliberately restricted supporting actors’ freedom. In line with Cnaan et al.’s (1996) assertion that volunteering is not only a free act but must contribute to the public good, coworkers justified pushing first responders to grow their skillset by referring to patient safety and public perceptions of crews’ competence. Perhaps this tendency is exacerbated because ambulance volunteering is a high-stakes role and seen as productive “work” more than as “serious leisure” (Stebbins, 2002).

Because volunteering requires the cost of engagement to exceed the rewards (Cnaan et al., 1996; Smith et al., 2017), coworkers accused thrill seekers, who avoided undesirable jobs, of “undermin[ing] volunteering from within” (Hustinx, 2010, p. 175). Volunteers benefited from the excitement of lights and sirens and public acclaim yet volunteering cost them little. Coworkers’ attempts to rein them in did not usually work. Drawing on Meijs and Brudney’s (2007) AAA approach, thrill seekers had the required *assets* but resisted applying them during boring or dirty organizational *assignments*. Generic appeals to teamwork were ineffectual because stepping back during routine calls did not undermine the public good as did lack of training.

Yet, fanning ideal workers’ initially unquenchable desire for excellence and progression down what paid staff considered a successful volunteer pathway could also be harmful. Paid staff expectations that the volunteer *assignment* be performed on par with paid staff from EMT level onwards pressured volunteers to increase their *availability* (number of shifts) and grow their *assets* (on-road skills and knowledge) through training and study (Meijs & Brudney, 2007). Nonetheless, exhaustion could lead the “bug” to peter out: Ideal workers who volunteered intensively (taking on all types of tasks) and extensively (signing up for multiple shifts) risked burnout (McNamee & Peterson, 2016) and, potentially, work–family conflict (Oelberger, 2019). As Bailey et al. (2019) highlight, although “individuals have an innate drive to seek out meaningful work to satisfy their inner needs, (…) this same drive can push them to harmful excesses” (p. 489). In the same way that subjective accounts of meaningfulness could be mistaken when volunteers were clinically underprepared, social accounts could be too, if volunteers were clinically savvy but overworked. These findings challenge the notion that positive freedom is always a stimulus for subjective flourishing and emancipation (Florian et al., 2019).

The study also contains practical insights for those who manage volunteers. First, NPOs need to use a variety of criteria apart from time commitment to identify “super”-volunteers. Second, because unique freedom pathways will likely require distinctive assignments, NPOs could develop a recruitment tool that identifies volunteers’ preferences. Finally, managers must establish clear guidelines surrounding minimum and maximum expectations. Thrill seekers may be better managed by appeals to organizational policies than through interpersonal persuasion; maximums may help ideal workers engage more sustainably.

## Limitations and Future Directions

Despite this study’s contributions, which include theorizing a more relational perspective of freedom and documenting freedom’s dark side, this study has several limitations that future research should address. First, this study employed a cross-sectional data collection strategy, which precluded charting longitudinal shifts in workers’ preferences or beliefs about freedom over time (e.g., could jaded, exhausted ideal workers metamorphose into supporting actors?). Second, the theoretical framework of positive and negative freedom (freedom *to* and freedom *from*, respectively) may have led me to overlook less visible forms of freedom present in the data, such as freedom *with*. Given the importance of positive interpersonal relationships for meaningfulness (Lips-Wiersma & Morris, 2017), studies need to focus on “who” helps workers experience meaning at work (Robertson et al., 2020). Future research could develop typologies of varied forms of freedom *and* relationality, in order to better nuance how workers experience meaningfulness.

Researchers could also assess how different types of workplace relationships and perceptions of fairness and workplace justice with coworkers, hierarchical superiors, supervisors, managers, leaders, and beneficiaries of one’s work shape the nature and experience of freedom and meaningfulness (Lips-Wiersma et al., 2020). When do relational concerns impel workers to put aside overly individualistic conceptions of freedom to build or preserve the relationship? Doing so will show not only when workers are relationally wrong about meaningfulness, but when they are right.

## Supplementary Information

Below is the link to the electronic supplementary material.

Supplementary file1 (DOCX 17 kb)

Supplementary file2 (DOCX 16 kb)

## Footnotes

1 In this text, I take freedom, autonomy, and liberty to be synonyms.

2 Double crewing requires the presence of two ambulance crew: one crew member drives while the other gives ongoing prehospital care and monitors changes in the patient’s condition on route to hospital.

3 An authority to practice (ATP) indicates what treatments ambulance crew can give at their current qualification level.

4 Fluid deployment refers to an organizational practice whereby crews are sent to “cover” another station because that crew is responding to a call. Fluid deployment enhances the ability to respond quickly to urgent local jobs.
