# Supplementary material for: Ideal Workers, Supporting Actors, or Thrill Seekers? How Coworker Demands Influence Ambulance Volunteers’ Experiences of Freedom and Meaningful Work
Source: Voluntas. 2024 Oct 22;36(1):32–42. doi: 10.1007/s11266-024-00690-3 (PMC11882614; doi:10.1007/s11266-024-00690-3)
Supplement: Supplementary file 1 — Supplementary file1 (DOCX 17 kb) [file 11266_2024_690_MOESM1_ESM.docx]

Supplementary File 1

## Interview Guide

1. How did you get involved with St John? Did you start as a volunteer or paid staff member?
2. What are your responsibilities at the station? In an ideal world, what would you like your responsibilities to include?
   1. Who do you report to?
   2. How regularly do you talk to him/her?
   3. How does this impact your role?
   4. What kind of support would you want?
3. What are your responsibilities on-road?
4. What is the role of paid staff/volunteers (depending on the role of the person interviewed)? What are they *not* responsible for?
5. What jobs should they do that they don’t do?
   1. How do you manage this?
   2. What are your expectations of paid staff/volunteers in general?
6. How do you decide who does what at the station?
7. How do you decide who does what on the ambulance?
8. Do you feel that tasks/responsibilities are fairly distributed within paid/volunteer crews? (Probe for what is “fair”). How do you feel about the decision-making process?
9. St John describes all ambulance officers (both paid and volunteer) as “health professionals” of equal status. What is your stance on this?

10. How would you describe your relationships with the other crew in this station? (Probe for specific examples). How could relationships be improved? (Probe for who would be responsible for improving relationships).
