# Supplementary material for: Ideal Workers, Supporting Actors, or Thrill Seekers? How Coworker Demands Influence Ambulance Volunteers’ Experiences of Freedom and Meaningful Work
Source: Voluntas. 2024 Oct 22;36(1):32–42. doi: 10.1007/s11266-024-00690-3 (PMC11882614; doi:10.1007/s11266-024-00690-3)
Supplement: Supplementary file 2 — Supplementary file2 (DOCX 16 kb) [file 11266_2024_690_MOESM2_ESM.docx]

Supplementary File 2

## An Example of the Coding Process

| **Raw data from interviews and fieldnotes** | **First-level codes** | **Second-level codes** | **Volunteer profile** |
| --- | --- | --- | --- |
| “I’ve got the bug and love it. And, so, that’s cool.”  “People here, they have to love it. To go through what they do on the road and back at the station sometimes, you’ve got to love it.”  “They absolutely love it, that’s what they like to do.”  “I like sitting here now, wondering what’s going to go off next and what I’m going to be going to. That excites me.”  “He is still at the stage where, you know, we might have a three-car accident tonight and he’s really quite excited by the whole thing.” | Having the bug  Loving ambulance work  Having a sense of the unknown  Feeling excited | Engaging in absorbing, stimulating, challenging work | Ideal Workers love their role because they find ambulance work interesting, exciting, and a source of growth. Their enthusiasm manifests by intense involvement in terms of the number of weekly shifts completed, a desire to take on more complex jobs, and commitment to further training. |
| “You have very clear intentions of where you’re heading and what you’re trying to achieve.”  “They want to be doctors on Day 1, completely hands on and in charge, leading jobs where they assess and treat patients to the level that they are capable of.”  “They put their hands up, they actually want to get involved more. They don’t want to sit back and wait: They want it now.”  “They’re chomping at the bit to get on the next courses, they’re chomping at the bit to do the first responder [course], chomping at the bit to be an EMT.”  “[Although] volunteers’ job is not St John (…) you’ll find there’s a lot of volunteers that do a lot of shifts here. If you look on the roster, you’ll see several people’s names occurring over and over again. They’re amazing –they love it, but they’re still *volunteers*, and that amazes me. You know, they give up like three or four [12-hour] shifts a week.” | Setting and attaining goals  Leading jobs  Assessing and treating patients  Getting involved  Wanting to do more  Pushing to start next-level training  Signing up for multiple shifts | Demonstrating eagerness to advance further by increasing quantity and quality of on-road experiences  Taking advantage of training |  |
